# Synthesis and Performance of Polycarboxylate Superplasticizer with Viscosity-Reducing and Low-Shrinkage Properties for Fair-Faced Concrete

**DOI:** 10.3390/ma17112685

**Published:** 2024-06-02

**Authors:** Wei Li, Chunxiang Qian, Chunyang Zheng, Haidong Jiang, Zhenxiao Yu, Zefan Wu, Zhuang Zhou

**Affiliations:** 1 School of Materials Science and Engineering, Southeast University, Nanjing 211189, China; aritlw@outlook.com (W.L.); cxqian@seu.edu.cn (C.Q.); zhouzhuang@seu.edu.cn (Z.Z.); 2 Jiangsu Arit New Materials Co., Ltd., Nanjing 211505, China; aritzcy@outlook.com (C.Z.); 4834567@163.com (H.J.)

**Keywords:** polycarboxylate-based water reducer, low-shrink admixtures, plastic viscosity

## Abstract

A low-shrinkage and viscosity-reducing polycarboxylate superplasticizer was synthesized with maleic anhydride (MAH), diethylene glycol monobutyl ether, and methoxypoly (ethylene glycol) methacrylate (MPEG_n_MA). The surface tension, early shrinkage, cement paste performance, and application performance of concrete made with the synthesized water-reducing admixture were tested. A series of experiments were conducted to determine the optimal range of plastic viscosity coefficients for producing high-quality, fair-faced concrete with minimal surface defects. These experiments utilized both the synthesized water-reducing admixture alone and in combination with other water-reducing agents. The results showed that the synthesized water-reducing admixture had an ideal molecular structure, as confirmed by the GPC spectrum. When added to an aqueous solution, it reduced the surface tension from 72.47 mN/m to 30.56 mN/m. The 72 h shrinkage value of concrete was reduced by 20.6% compared with that of the conventional control group, effectively reducing shrinkage and adjusting the viscosity of the concrete mixture. Additionally, the influence of the plastic viscosity coefficient on the apparent voids in fair-faced concrete was investigated. This study revealed that when the plastic viscosity coefficient was between 5 and 10 Pa·s, the apparent void grade of the fair-faced concrete was simultaneously excellent and good. This water-reducing admixture helped prevent surface cracking and voids in fair-faced concrete, making it a suitable choice for producing high-quality fair-faced concrete surfaces.

## 1. Introduction

Fair-faced concrete is a kind of concrete that directly utilizes the natural texture of concrete after molding as the final decorative effect [1], being also known as decorative concrete [2]. Compared with ordinary concrete structures, fair-faced concrete structures have a clean surface, no chiseling or damage, and a uniform color. These constitute a new type of concrete structure that is not only aesthetically pleasing but also environmentally friendly, as evidenced by low cement usage, low dust generation (a large amount of dust is generated during the subsequent decorative treatment of ordinary concrete), and better durability [3,4,5]. Due to the tremendous advantages of fair-faced concrete over traditional concrete, many large-scale constructions now tend to choose fair-faced concrete for design [6,7]. Although fair-faced concrete has been introduced in China for thirty years, it has developed rapidly in recent years with the deepening of the concepts of saving society and sustainable development. However, the appearance defects in fair-faced concrete, such as voids and cracking, bother its users.

The “fair-face” characteristics of fair-faced concrete placer higher requirements for surface quality. However, there are a lot of problems in its application in engineering, among which the surface quality problem is the one that needs to be solved urgently. Surface quality problems mainly refer to color differences and voids. The current methods for dealing with voids from the construction point of view include adopting an appropriate vibration time, reducing the thickness of each pour and construction, using a permeable framework and mold-releasing agent, choosing effective defoamer, and repairing the voids by chiseling the surface of the voids in an appropriate amount, and then smoothing it with cement paste [8,9,10,11]. Wallevik et al. [12] performed a statistical examination of the rheological characteristics of various concrete types and applications by analyzing the existing literature and incorporating their own experimental findings. Specifically, they investigated the suitable ranges of these parameters for self-compacting fair-faced concrete to achieve good flowability and an excellent surface appearance. However, these methods require experience and lack reproducibility. 

The treatment of voids at the raw material stage involves adjusting the workability of the concrete, such as via adjusting the viscosity to the appropriate range, which is commonly achieved with the use of water-reducing agents [13,14,15]. Due to the use of high-quality admixtures and water-reducing agents, the number of cementitious materials in fair-faced concrete is larger, and the water–cementitious ratio is lower than that of ordinary concrete. When high-strength or ultra-high-strength concrete is used due to engineering needs, concrete shrinkage can easily lead to concrete cracking, affecting the surface quality of fair-faced concrete. The main methods to reduce the shrinkage of concrete are the incorporation of fiber, a shrinkage reducer, and an expansion agent. However, these methods have several limitations: When the amount of expansion agent is too low, it cannot achieve the desired effect; when the amount of mixing is too high, it may lead to overexpansion and cracks or make the existing cracks become larger [16]; fiber has a certain effect of reducing shrinkage, and the cost is acceptable, but it is not well compatible with the concrete. Some shrinkage-reducing products in the market have a good shrinkage-reducing effect, but the required mixing amount and the unit price are high, so that the cost of concrete rises greatly [17,18]. Therefore, it is of great practical significance to research and develop low-shrinkage and viscosity-reducing polycarboxylic acid water-reducing agents.

The high performance of polycarboxylic acid water-reducing agents is realized by selecting monomers with different structures and functions, designing polycarboxylic acid water-reducing agents with specific molecular structures, and choosing the best combination of main chain polymerization degree, side chain length, and functional group types. And, according to experimental results, a polycarboxylic acid water-reducing agent with the ability to reduce shrinkage and adjust viscosity was prepared to solve the problems of cracking and voids on the surface of fair-faced concrete. Meanwhile, using the synthesized polycarboxylic acid water-reducing agent in combination with commercially available polycarboxylic acid lipid-based high-efficiency water-reducing agents and polycarboxylic acid ether-based water-reducing agents, a full-factor level test was carried out on the highly robust parameter intervals within the ranges of the initial air content of 2–3% and of the particle size of the coarse aggregate of 5 mm to 25 mm to explore the influence of the coefficient of plastic viscosity on the apparent void indices of the fair-faced concrete. 

In this study, a low-shrinkage, viscosity-reducing polycarboxylic acid-based water reducing agent was synthesized by using methoxy polyethylene glycol methacrylate macromonomer, maleic anhydride monomer, and diethylene glycol monobutyl ether as raw materials through a redox initiation system, aiming to reduce the shrinkage of the concrete. At the same time, the effects of the average molecular weight, side chain length, and methacrylic acid content of the synthesized water-reducing agent on the viscosity of concrete were analyzed and investigated through gel permeation chromatography (GPC) testing experiments. Additionally, a series of experimental tests, including surface tension measurements of the water-reducing agent in aqueous solutions, its impact on the fluidity of cement pastes, and its performance in freshly mixed concrete, were also conducted. An orthogonal experimental design was employed to explore the influence of plastic viscosity coefficients on the apparent voids in fair-faced concrete, and we further discussed the role of the low-shrinkage, viscosity-reducing polycarboxylate water-reducing agent in fair-faced concrete.

## 2. Materials and Methods

### 2.1. Raw Materials

Raw materials used included (1) methacrylic acid-polyethylene glycol monomethyl ether ester macromonomer (MPEG_n_MA). The molecular weight of the polyethylene glycol monomethyl ether ester was 750, 2000, and 3000 (expressed as MPEG_16_MA, MPEG_45_MA, and MPEG_67_MA, respectively), produced by Liaoning Kolon Fine Chemical Co. (Liaoyang, China) (2) Cellulose ether MHPC500PF was used as a tackifier, produced by Shanghai Shangnan Trading Co. (Shanghai, China) (3) Diethylene glycol monobutyl ether, maleic anhydride (MAH), sodium methallyl sulfonate (SMAS), hydrogen peroxide, and sodium hydroxide, K12, all analytically pure, were purchased from Sinopharm Chemical Reagent Corporation (Shanghai, China). Commercially available Vitamin C was also used.

### 2.2. Experimental Materials

The chemical and mineral compositions of P·I 42.5 benchmark cement are shown in Table 1.

The medium sand had a fineness modulus of 2.6, a density of 2551 kg/m^3^, and a bulk density of 1461 kg/m^3^. The crushed stone was continuous graded gravel with a particle size ranging from 5 mm to 20 mm. Among them, 5 mm to 10 mm accounted for 40%, 10 mm~20 mm accounted for 60%, needle and flake particles accounted for less than 5% of the total. The compact void ratio was less than 40%, the mud content was less than 0.5%, the density was 2630 kg/m^3^, and the bulk density was 1540 kg/m^3^. The water used for the experiment was tap water.

### 2.3. Main Instrumentation

The thermostatic water bath HH-S1 was produced by Jintan Medical Instrument Factory (Changzhou, China). The electric mixer, JJ-A, was produced by Jiangsu Jintan Ronghua Instrument Manufacturing Co., Ltd. (Changzhou, China) Cement paste mixer NJ-60A was produced by Wuxi Jianyi Instrument Machinery Co., Ltd. (Wuxi, China). The mandatory mixer for the concrete test was a HJW60, produced by Wuxi Jianyi Instrument Machinery Co., Ltd. (Wuxi, China). The microcomputer-controlled pressure testing machine was a WHY2000, produced by Shanghai Hualong Testing Instrument Co., Ltd. (Shanghai, China).

### 2.4. Synthesis of Low-Shrinkage, Viscosity-Reducing Water-Reducing Agent

- For the synthesis of low-shrinkage functional monomer, diethylene glycol monobutyl ether, maleic anhydride, and catalyst were directly added into a four-necked bottle, reacted at 100–140 °C for 5 h, distilled under reduced pressure, cooled, and discharged for spare material.
- For the she synthesis of low-shrinkage, viscosity-reducing polycarboxylate water-reducing agent, we weighed hydrogen peroxide, sodium methallyl sulfonate, and water and added them to a three-necked flask containing the macromonomer MPEG_n_MA, which was stirred and heated in a water bath for 15 min. The mass ratio of the components was hydrogen peroxide: sodium methallyl sulfonate: macromonomer MPEG_n_MA = 1:4:120. Simultaneously, we added components A and B drop-wise. Component A was the low-shrinkage functional monomer, consisting of 12 g of acrylic acid and 28 g of water. Component B was the Vc aqueous solution, consisting of 0.2 g of Vc and 46 g of water. With a drop rate of 0.22 mL/min, the addition of components A and B was completed in 3 h and 3.5 h, respectively, which was followed by maintaining the reaction temperature for 1 h. After cooling, the pH value of the reactants was adjusted to neutral with NaOH solution at a concentration of 40%, and the mother liquor of polycarboxylic acid water-reducing agent obtained at the end of the reaction was loaded into a dialysis bag (the molecular weight of retention was 5000), and the volume of the water-reducing agent accounted for one-fifth of the capacity of the dialysis bag. The water was changed 4–6 times per day, and the required low-shrinkage and viscosity-reducing polycarboxylic acid water-reducing agent could be obtained after one week of dialysis. After one week of dialysis, the required low-shrinkage and viscosity-reducing polycarboxylic acid water-reducing agent was produced.

### 2.5. Performance Testing and Characterization

#### 2.5.1. Gel Permeation Chromatography (GPC) Analysis

The molecular weights and distributions of the synthesized samples were determined using a Waters 1515 gel permeation chromatograph (Waters, Milford, MA, USA). The mobile phase was 0.1 mol/L NaNO_3_ solution at a flow rate of 1.0 mL/min, and the stationary phase was a gel-like porous filler.

#### 2.5.2. Surface Tension Test

A surface tension test was carried out in accordance with GB/T 8077-2012 [19] to determine the surface tension of the solution doped with polycarboxylic acid water-reducing agent; Dataphysics OCA40 microtype surface contact angle tester (Filderstadt, Germany) was used, which had a measurement range of 0.01~2000 mN/m, a measurement accuracy of ±0.01 mN/m, and a contact angle measurement range of 0~180°.

#### 2.5.3. Concrete Shrinkage

Referring to the contact shrinkage test method in GB/T 50082-2009 [20], the test was carried out. A PVC pipe with a diameter of 100 mm and a height of 515 mm was used as a mold to form the concrete specimen, and the specimen was placed in an environment with a temperature of 20 ± 2 °C and a relative humidity of 60 ± 5% immediately after forming. A small piece of glass was placed on the upper surface of the specimen, and the probe of the automatic deformation collector was gently pressed against the glass. The automatic shrinkage collector was turned on to collect the shrinkage value in the 72 h after the concrete was first set.

#### 2.5.4. Net Cement Paste Test

The fluidity of cement mortar and its retention performance were determined according to GB/T 8077-2012. The water–cement ratio was 0.29, and the water-reducing agent dosage was 0.12%.

#### 2.5.5. Concrete Tests

For concrete extensibility, T_50_ test, and V funnel determination, we referred to the relevant requirements in JGJ/T 283-2012 [21].

#### 2.5.6. Experiment on Controlling the Viscosity of Concrete and Its Impact on the Appearance of Fair-Faced Concrete

(1) Raw material

The raw materials used in the test were PO 42.5 ordinary Portland cement produced by Nanjing Conch Cement Co., Ltd. (Nanjing, China), which had a density of 3.16 g/cm^3^; PO 42.5 ordinary Portland cement produced by Nanjing Jiangnan Xiaoyetian Cement Co., Ltd. (Nanjing, China), which had a density of 3.13 g/cm^3^; Class I fly ash produced by Jiangsu Changshu Power Generation Co., Ltd. (Changshu, China); S95-grade mineral powder produced by Lianfeng Steel Zhangjiagang Co., Ltd.(Zhangjiagang, China); coarse sand from Dongting Lake in Hubei, sieved to 4.75 mm; stone in the range of 5 mm to 25 mm; the polycarboxylate-based high-efficiency water-reducing agent (PSA) with a water-reducing rate of 30%, provided from Sika (China) Co., Ltd. (Suzhou, China); the polycarboxylate ether-based high-efficiency water-reducing agent (PSB) with a water-reducing rate of 30%; drinking tap water used for adjusting water content. The chemical composition of the microbial antiefflorescence agents is shown in Table 2.

(2) Specimen preparation

The investigation covered the concrete strength grades commonly used in construction, and, after a comprehensive comparison, it was decided to conduct experiments using three strength grades: C40, C50, and C60. Cross-preparation experiments for fair-faced concrete were conducted using the aforementioned raw materials and the three strength grades to explore the highly robust parameter control range of the apparent voids in fair-faced concrete. The specific experimental mix proportions are shown in Table 3.

**Figure 1:**
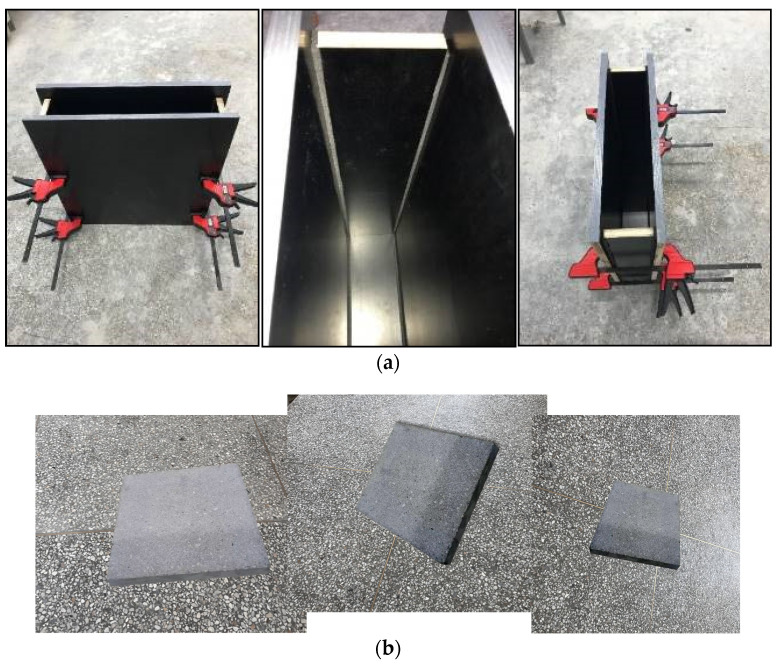
(**a**) Fair-faced concrete module; (**b**) prepared concrete specimens.

In order to conduct a large number of experiments and facilitate the observation of the apparent quality of the fair-faced concrete specimens, wooden molds with dimensions of 500 mm × 100 mm × 500 mm (length, width, and height) were designed. Sponge strips were used to seal the gaps between the molds. Each mold was equipped with four quick-release clips. Before each test, a brush was used to evenly apply mold-release agent to the surface of the molds. When demolding, the quick-release clips were removed, as illustrated in Figure 1a,b.

(3) Experimental design

Due to the various factors and levels involved in this experiment, it was necessary to use a rational experimental design method to more effectively and reasonably explore the material’s parameter range with high robustness for controlling the apparent voids in fair-faced concrete. The parameters of the raw experimental materials that were adjusted included the type of cement, type of water-reducing agents, powdered content of fine aggregates, and angular content of coarse aggregates. This was an orthogonal experiment design. 

Orthogonal experiments, also referred to as orthogonal array-based designs or Taguchi designs, are a methodical and economical approach to conducting experimental studies. These experiments aim to evaluate the simultaneous impact of multiple factors on a response variable of interest. The fundamental objective of an orthogonal experiment is to examine the effects of various factors on a response variable while minimizing the required number of experimental trials. This efficiency is achieved by strategically selecting a subset of all possible factor–level combinations, known as an orthogonal array. The orthogonal array represents a carefully curated set of factor–level combinations chosen to maintain the independence and orthogonality among the factors under investigation. By employing an orthogonal array, researchers can study the impacts of multiple factors simultaneously while significantly reducing the experimental workload compared to that of traditional full-factorial designs. This approach allows for a comprehensive understanding of factor effects and interactions while optimizing resources and minimizing experimental costs [22,23,24,25,26].

From the perspective of orthogonal experiments, the adjustment of raw materials involved the variation of four factors at three levels, and the number of level combinations in the experiment is shown in Table 4. The evenly distributed level combinations were A_1_B_1_C_1_D_1_, A_1_B_2_C_2_D_2_, A_1_B_3_C_3_D_3_, A_2_B_1_C_2_D_3_, A_2_B_2_C_3_D_1_, A_2_B_3_C_1_D_2_, A_3_B_1_C_3_D_2_, A_3_B_2_C_1_D_3_, and A_3_B_3_C_2_D_1_. In addition, after obtaining suitable ranges for different material parameters, the apparent quality of the prepared fair-faced concrete was verified through experiments.

It was found that all 4 main effects could be fully evaluated using the 9 combinations in Table 4, without being affected by interactions. Furthermore, some two-factor interaction effects, such as AB, AC, and BC, could also be estimated within these 9 combinations. Moreover, these 9 combinations were orthogonal across all columns (factors), ensuring the unbiasedness and effectiveness of the experimental results. At the same time, this was the most efficient minimum number of experimental runs for studying 4 factors at 3 levels.

(4) Parameter measurement method of adjustment and determination of yield stress and plastic viscosity coefficient

The initial yield stress and plastic viscosity coefficient of water–cement paste were determined using a Brookfield RST rotational viscometer, provided by Ametek Trading (Shanghai) Co., Ltd. (Shanghai, China). To ensure the accuracy of the data measurement and to avoid damaging the rotor, the coarse aggregate in the paste needed to be drained before the measurement.

The program for the concrete rheological characteristics tester was designed with four stages:

① Slow rotation stage: speed of 0.1 rpm, duration of 60 s;

② Acceleration stage: duration of 100 s, speed in 10 gradients, each gradient increasing by 10 rpm, duration of 10 s for each gradient;

③ Constant-speed stage: duration of 60 s, speed of 100 rpm;

④ Deceleration stage: duration of 100 s, speed in 10 gradients, each gradient decreasing by 10 rpm, duration of 10 s for each gradient. The measured data were fitted using the Bingham model [27]. The Bingham rheological equation is as follows:

τ = τ0 + ηp·γ(1)

where τ is the shear stress experienced by the Bingham fluid in Pa, τ0 is the yield stress of the Bingham fluid in Pa, ηp is the plastic viscosity of the Bingham fluid in Pa·s, and γ is the shear rate of the Bingham fluid in the flow state in s^−1^.

The initial yield stress and plastic viscosity coefficient of the paste were adjusted by the dosage of water-reducing agent, with dosages of 0%, 0.3%, 0.6%, 0.9%, 1.2%, and 1.5%.

(5) Quantitative measurement and evaluation methods of the apparent voids in fair-faced concrete

The surface voids were used to comprehensively describe the void condition on the surface of water-cured concrete, with quantitative evaluation criteria shown in Table 5.

The method of quantitatively measuring and evaluating the apparent voids in fair-faced concrete using a 20-megapixel digital camera for image acquisition was as follows: The surface size of the specimen was 500 mm × 500 mm, and the area acquired was relatively small, so the distance of the lens from the specimen surface was set to 1 m. During the acquisition, adequate ambient light was ensured, a scale with an accuracy of 0.1 mm was placed in the image capture area, and the lens was positioned vertically to the specimen surface at its center.

**Figure 2:**
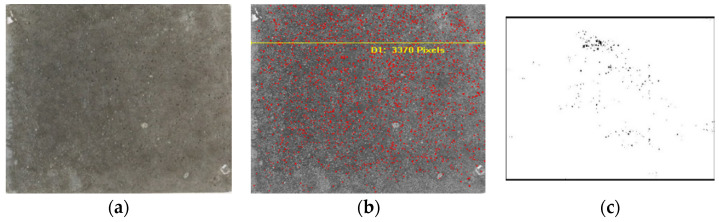
(**a**) Image import; (**b**) dimensional calibration; (**c**) pore determination.

The software: Image-Pro-Plus 6.0 was used for surface analysis, and the steps were as follows:

- Image capture: Imported the photo of the fair-faced concrete surface into the software, as shown in Figure 2a.
- Dimensional calibration: Clicked on the measurement calibration tool and dragged the ruler to two scales in the image to obtain the number of pixels between the two scales. The measured data were then converted to represent the actual measured length. Utilized Image-Pro-Plus 6.0 to calibrate the dimensions of the fair-faced concrete image, input the actual dimensions of the fair-faced concrete, and annotated the dimensions of the fair-faced concrete structure and the voids on its surface in the software, as shown in Figure 2b.
- Pore determination: For image grayscale processing and selection of the grayscale value of the pores, since the voids in the photo were black, the “automatic bright objects” option was selected. The purpose of binarization is to present two extreme colors (black or white) for the holes and hole walls in the image to improve measurement identification and calculation accuracy. When conducting grayscale binarization processing, the grayscale value of the voids was selected to distinguish between the concrete and the apparent voids, as shown in Figure 2c.
- Data export: Clicked on “measurement data” to view and output collected results, including surface void area percentage and other data, and clicked “statistics” to summarize all data.

The surface void area ratio was directly exported using Image-Pro-Plus 6.0. The sample surface was partitioned into 25 equal parts, and the surface void area ratio of each subregion was measured.

**Figure 3:**
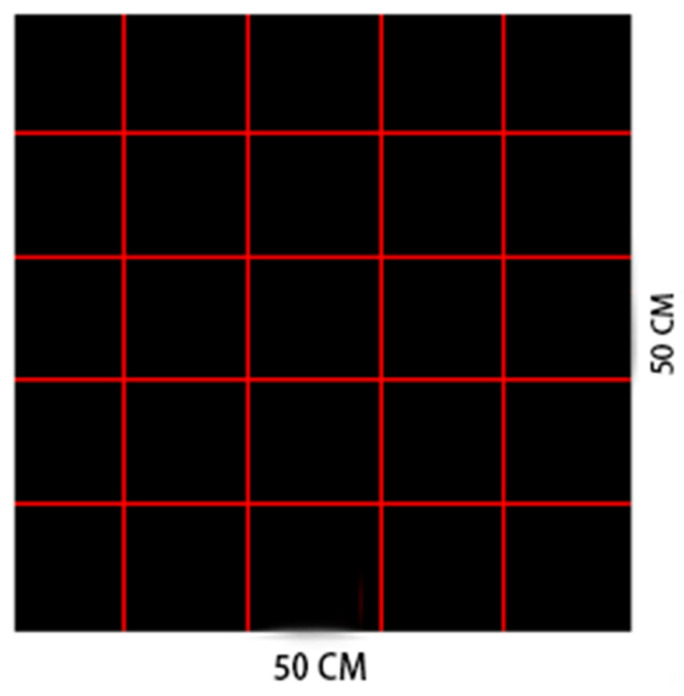
Example of surface division in test piece.

A larger result value indicates a more uneven pore distribution. Since the scale of specimen was 500 mm, the edge length of each subarea was set as 100 mm. The specimen surface division is shown in Figure 3, which illustrates the result of dividing the specimen surface into 25 parts.

Based on the highly robust parameter range (initial gas content of 2–3% and coarse aggregate size of 5 mm to 25 mm), experiments were conducted. Four-level combinations, namely, A_2_B_3_C_1_D_2_, A_3_B_1_C_3_D_2_, A_3_B_2_C_1_D_3_, and A_3_B_3_C_2_D_1_, were selected from the full-factorial design. Each level combination was tested using three concrete strength grades: C40, C50, and C60. Within each strength grade, tests were conducted with water-reducing agents at dosage levels of 0%, 0.3%, 0.6%, 0.9%, 1.2%, or 1.5%. For each level combination, a total of 18 sets of experiments were conducted. After each experiment, a portion of the fresh concrete mixture was taken to separate out the coarse aggregate in order to obtain mortar for quick measurements of plastic viscosity coefficient and initial yield stress values. The apparent stomatal data were analyzed using Image-Pro-Plus 6.0 and then imported into Origin 2019 for plotting.

## 3. Results and Discussion

### 3.1. Molecular Weight and Its Distribution of Low-Shrinkage, Viscosity-Reducing Polycarboxylic Acid Water-Reducing Agent P(MAH-MPEG_n_MA)

**Figure 4:**
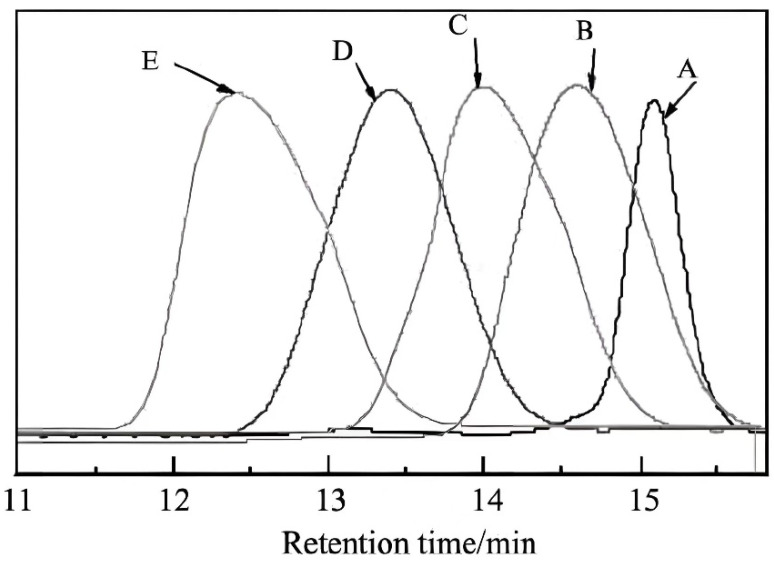
GPC trace of P(MAH-MPEG_n_MA)_m_.

In order to investigate the effects of the average molecular weight, side chain length, and methacrylic acid content of the viscosity-reducing water reducers on the viscosity of the concrete, two sets of experiments were designed: (1) using MAH and MPEG_16_MA with different MAH:MPEG_16_MA ratios and the same polyether side chain, MPEG_16_MA, to synthesize P(MAH_3.5_-MPEG_16_MA)_5_, P(MAH_4_-MPEG_16_MA)_9_, and P(MAH_4.5_-MPEG_16_MA)_12_; (2) using different polyether side chains (MPEG_16_MA, MPEG_45_MA, and MPEG_67_MA) and the same MAH:MPEG_n_MA ratio of MAH- and MPEG_16_MA-synthesized P(MAH_4_-MPEG_16_MA)_9_, P(MAH_4_-MPEG_45_MA)_9_, and P(MAH_4_-MPEG_67_MA)_9_. The results obtained from the experiments are shown in Table 6 and Figure 4. The feed ratio in the table refers to the mass ratio of the two monomers.

The values of the GPC results in Table 6 correspond to the peak shapes in Figure 4. In Figure 4, the horizontal axis represents the retention time, and the vertical axis reflects the relative concentration in the GPC test results. The width of the peak reflects the breadth of the molecular weight distribution, from which it can be seen that the GPC spectra of the generated products mainly concentrated in a very narrow range with ideal structures.

### 3.2. Surface Tension Test of Low-Shrinkage, Viscosity-Reducing Polycarboxylic Acid Water-Reducing Agent in Aqueous Solution

The Wilhelmy method was used to determine the surface tension of the low-shrinkage and high-slump-proof polycarboxylic acid water-reducing agent solutions at concentrations of 1.0%, 2.0%, 3.0%, 4.0%, 5.0%, 6.0%, 7.0%, 8.0%, 9.0%, and 10%. The same group of solutions was measured three times consecutively, and the average value was taken.

**Figure 5:**
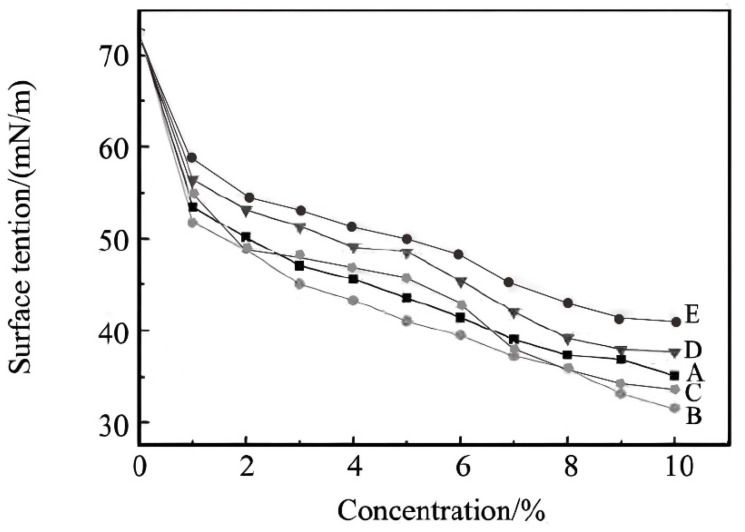
The surface tension of P(MAH-MPEG_n_MA)_m_ at different concentrations.

The result of the surface tension for each concentration is given in Figure 5. The horizontal axis represents the concentrations of the five low-shrinkage and viscosity-reducing polycarboxylate superplasticizers A, B, C, D, and E listed in Table 2, with a maximum value of 10%. The vertical axis represents the corresponding surface tension measured for each superplasticizer at the given concentration. As can be seen from Figure 5, the surface tension of pure water without any additive was 72.47 mN/m. When P(MAH_4_-MPEG_45_MA)_9_ was added at a concentration of 10%, the surface tension of the solution was 30.56 mN/m. It can be seen that the surface tension decreased significantly after the addition of the polycarboxylic acid water-reducing agent, which had a great influence on the improvement in the dispersibility and dispersion retention performance of the cement particles. From the overall trend of the figure, it can be seen that with the increase in the concentration of polycarboxylic acid water-reducing agent, the decrease in surface tension was P(MAH_4_-MPEG_16_MA)_9_ > P(MAH_4.5_-MPEG_16_MA)_12_ > P(MAH_3.5_-MPEG_16_MA)_5_ > P(MAH_4_-MPEG_45_MA)_9_ > P(MAH_4_-MPEG_67_MA)_9_. The main chain of P(MAH_4_-MPEG16MA)_9_ is long, and the hydrogen atoms of carboxylic acid group in the main chain can easily join with the oxygen atoms in the branched chain due to hydrogen bonding, so that the interaction between the macromolecular chains can be improved. The surface tension of the synthesized polycarboxylic acid water-reducing agent also increased due to the increase in the interaction force between the macromolecular chains and the increase in the viscosity of the solution caused by the large degree of aggregation. The spatial site resistance provided by MPEG_67_MA in the branched chain of P(MAH_4_-MPEG_67_MA)_9_ was much larger than that provided by MPEG_45_MA and MPEG_16_MA, so the surface tension declined the most slowly with the increase in the concentration of water-reducing agent, and the overall decrease was smaller. The low surface tension of viscosity-reducing polycarboxylic acid water-reducing agent reduced the solid–liquid interfacial energy of the cement particles, and, at the same time, it easily formed a lot of tiny bubbles in the fresh concrete, which could isolate cement particles and was conducive to the adsorption of the water-reducing agent on the cement particles, so as to disperse and stabilize the cement particles.

### 3.3. Concrete Shrinkage

**Figure 6:**
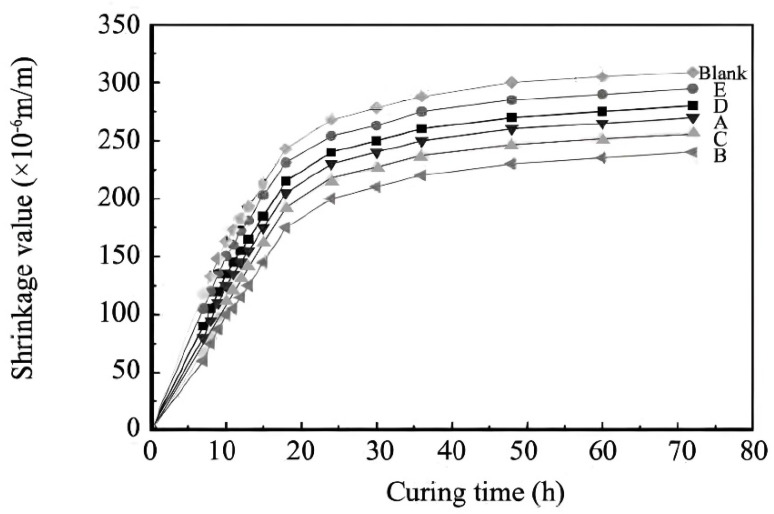
The result of early-age shrinkage of concrete.

The shrinkage values of the concrete specimens from the initial setting to 72 h are shown in Figure 6. The 72 h shrinkage values of the concrete doped with B and PC-2 water-reducing additives were 301 × 10^−6^ m/m and 340 × 10^−6^ m/m, respectively, which were 75% and 94.5% of those of the baseline concrete. The 72 h shrinkage value of PC-1-added concrete was 20.6% lower than that of PC-2-added concrete, which effectively ensured the safety of the project.

When different low-shrinkage, viscosity-reducing polycarboxylic acid water-reducing agents were incorporated, the shrinkage values significantly decreased, which was attributed to the fact that the diethylene glycol monobutyl ether molecular structure contains a water-repellent group, methyl, which improved the hydrophilic lipophilicity value (HLB) of the water-reducing agent’s molecular structure. The largest decrease was in the case of P(MAH_4_-MPEG_45_MA)_9_, and the smallest decrease was observed in the case of P(MAH_4_-MPEG_67_MA)_9_. The main reason for the reduction in the shrinkage of the low-shrinkage and viscosity-reducing polycarboxylic acid water-reducing agents was that the incorporation of low-shrinkage functional monomers greatly reduced the surface tension of concrete’s gas–liquid interfaces and improved the pore structure of the hardened cement paste, thus slowing down the evaporation of water from the internal pores and reducing the capillary tension due to the capillary pore loss of water, thus realizing the low-shrinkage effect. The results and the surface tension were highly consistent.

### 3.4. Effect of Low-Shrinkage, Viscosity-Reducing Polycarboxylic Acid Water-Reducing Agent on the Flow Properties of Net Mortar

The average molecular weight, side chain length, and maleic anhydride content of low-shrinkage, viscosity-reducing water reducers are important parameters of the reaction, which directly determine the initial flow and flow retention capacity of concrete. The results of the effect of different low-shrinkage and viscosity-reducing water-reducing agents on the flow properties of the cement mortar are shown in Table 7.

As can be seen from Table 7, when the side chain length was different and the ratio of MAH: MPEG_n_MA was 4:1, the initial flow and flow retention capacity was in the following order: P(MAH_4_-MPEG_16_MA)_9_ > P(MAH_4_-MPEG_45_MA)_9_ > P(MAH_4_-MPEG_67_MA)_9_, which indicates that when the density of the same side chain is different from that of the side chain lengths, the thickness of the water layer film in the concrete becomes gradually thinner, which can release more free water, with the net mortar showing excellent initial flow and flow retention capacity. This shows that (1) with the same side chain density and different side chain lengths, the thickness of the water film formed in the concrete is theoretically thinner, and more free water can be released, which lead to excellent initial flow and flow retention ability; (2) with the same side chain density and different side chain lengths, the molecular weight gradually increases, the water retention performance is increased, and the molecular weight and the side chain gradually increase, so that the carboxylic acid group content also increases, and the number of hydrogen bonds that can combine water with it increases, which in turn bind a certain amount of free water and affect the dispersion and slump-proofing performance. When the ratio of MAH: MPEG_n_MA was 4.5:1, the length of the side chain was certain, and the density of the side chain was lower than that when there were more adsorption groups. A large number of adsorption groups on the molecules of the water reducing agent may have been adsorbed on the cement particles, but the density of the side chain was too low to have enough repulsion, so that the effect of water reduction was generally achieved. When the ratio of MAH: MPEG_n_MA was 3.5:1, the length of side chain was certain, and the density of side chain was higher with fewer adsorption groups. This may have been due to the insufficient number of adsorption groups; coupled with the high density of side chains, it was difficult to realize spatial site resistance, so that the net slurry flowability and retention of performance were worse.

### 3.5. Effect of Low-Shrinkage and Viscosity-Reducing Polycarboxylic Acid Water-Reducing Agent on the Application Performance of Fresh Concrete

For the calculation and testing of the concrete mix ratio, we referred to GB 55008-2021 [28] and JGJ/T 283-2012 [21]. The amount of cement selected for this test was 520 kg/m^3^, and the sand rate was 48%. The water-reducing agents used in the test were 0.6%, 0.8%, 1.0%, and 1.2% of the mass of cement. The test ratios are shown in Table 8, and the results are shown in Table 9.

As can be seen from Table 9, when the amount of low-shrinkage and viscosity-reducing polycarboxylic acid water-reducing agent was 0.6~1.0%, the fluidity of the concrete increased rapidly from the time of T_50_ in a V-shaped funnel. When the dosage exceeded 1.0%, the growth in concrete fluidity slowed down and tended to be constant, and the concrete segregation phenomenon occurred. After comprehensive comparison, 1.0% was determined as the best dosage of the water-reducing agent. The good flow performance of the low-shrinkage and low-viscosity superplasticizer is attributed to its structure. On the one hand, the introduction of more hydrophobic groups to its molecular structure reduces its surface tension. For example, the introduction of polyether macromolecules into its side chain and the introduction of diethylene glycol monobutyl ether into its main chain reduce its HLB value, and the reduction in HLB value reduces the amount of bound water it generates with water. On the other hand, the performance of a water reducer also depends on the individual molecular efficacy and the number of molecules. The average molecular weight of P(MAH_4_-MPEG_16_MA)_9_ is only one-third that of ordinary polycarboxylic acid superplasticizer, so it has a higher degree of freedom in free water, is more able to stretch the molecular chain of the superplasticizer, and can be quickly adsorbed on the surface of the concrete in large quantities, so that the concrete has low viscosity and high fluidity.

### 3.6. Relationship between Surface Porosity Quantity and Plastic Viscosity Coefficient

**Figure 7:**
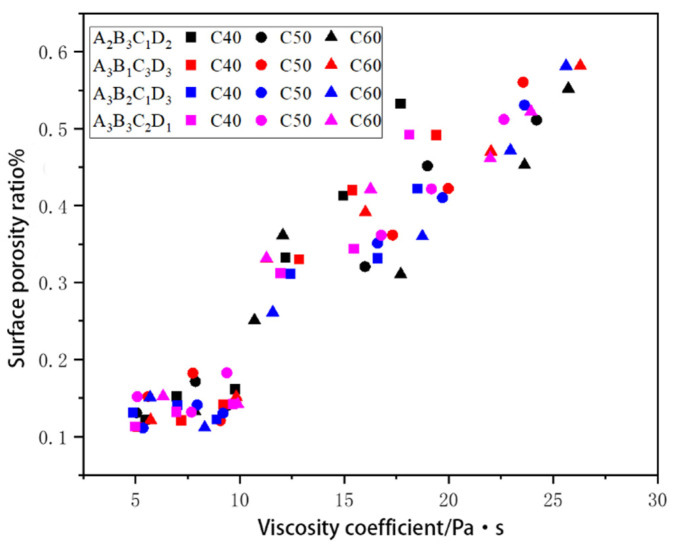
Influence of the plastic viscosity coefficient on the apparent voids in fair-faced concrete in four-level combination experiments.

The results of Experiment 2.5.6 is shown in Figure 7. The X-axis in the figure represents the change in the initial air content, while the Y-axis represents the variations in the surface void area ratio. The figure uses black, red, green, and blue to represent the four levels A2B3C1D2, A3B1C3D2, A3B2C1D3, and A3B3C2D1, and square, circle, and triangle are used to represent the three concrete strength grades C40, C50, and C60.

When analyzing the three plots in Figure 7, it can be observed that with the increase in the plastic viscosity coefficient, the scatter plot of the surface void area ratio shows a trend of nearly linear growth, remaining below 0.2% when the plastic viscosity coefficient is between 5 and 10 Pa·s. Additionally, it can be seen that with the increase in the concrete strength grade, the corresponding values of the surface void area ratio also increase.

A comprehensive view of the three plots in Figure 7 indicates that under different raw materials and mixtures, when the plastic viscosity coefficient was between 5–10 Pa·s, the apparent voids of the concrete were simultaneously in the excellent and good rating levels. At this point, the obtained level of apparent voids in the concrete was comparatively excellent.

## 4. Conclusions

In this study, based on the molecular design principle, a low-shrinkage, viscosity-reducing polycarboxylic acid-based high-performance water-reducing agent was synthesized via free radical co-polymerization, and the following main conclusions were drawn from this study:

(1) Methyl polyethylene glycol methacrylate macromonomer (MPEG_n_MA) and methacrylic acid (MAA) were used as the main raw materials to synthesize a viscosity-reducing polycarboxylate water-reducing agent. The molecular weight and its distribution in the synthesized product were characterized by GPC. The GPC spectrum showed that the formed polymer structure was relatively ideal.
(2) Experiments were conducted to test the surface tension of the water reducing agent in aqueous solutions, its effect on the fluidity of cement pastes, and its performance in freshly mixed concrete. The surface tension test revealed that when the concentration of P(MAH_4_-MPEG_45_MA)_9_ was 10%, the surface tension of the solution decreased significantly from 72.47 mN/m to 30.56 mN/m. The 72 h shrinkage value of concrete was reduced by 20.6% compared to that of the conventional control group. In the cement paste fluidity experiment, it was found that when the MAH:MPEG_n_MA ratio was 4:1, P(MAH_4_-MPEG_16_MA)_9_ maintained an optimal side chain density and number of functional groups, resulting in the best initial fluidity and fluidity retention ability. The testing of freshly mixed concrete showed that using a 1.0% dosage of the low-shrinkage, viscosity-reducing polycarboxylate water-reducing agent yielded the best results.
(3) The influence of the plastic viscosity coefficient on the apparent voids of fair-faced concrete was investigated through orthogonal combination experiments, which determined a numerical interval with higher robustness. Specifically, when the plastic viscosity coefficient was between 5 and 10 Pa·s, the apparent void grade of the fair-faced concrete was simultaneously excellent and good.

Overall, this low-shrinkage, viscosity-reducing type water-reducing agent has better viscosity-reducing ability than ordinary commercially available polycarboxylic acid water-reducing agent, producing concrete with excellent low-shrinkage and fluidity performance, and has better application prospects in the field of fair-face concrete considering the relevant conclusions above.

## Figures and Tables

**Table 1:** Chemical and mineral compositions of reference cement (wt%).

SiO_2_	A_2_O_3_	F_2_O_3_	CaO	MgO	SO_3_
22.92	4.38	2.88	66.14	1.94	0.32
N_2_Oeq	f-CaO	C_3_S	C_2_S	C_3_A	C_4_AF
0.73	0.62	58.59	21.57	6.59	8.69

**Table 2:** Chemical composition of microbial antiefflorescence agent.

SO_3_	Na_2_O	CaO	P_2_O_5_	MgO	Cl	K_2_O	Sum/%	Other/%
28.36%	22.55%	21.48%	0.957%	0.674%	0.538%	0.524%	75.083	Spore nutrients

**Table 3:** Mix proportion of fair-faced concrete for three strength grades.

Strength Grade	Water-Cement Ratio	Water/kg·m^−3^	Cement/ kg·m^−3^	Fly Ash/ kg·m^−3^	Mineral Powder/ kg·m^−3^	Sand/ kg·m^−3^	Stone/ kg·m^−3^	Water Reducing Agent/%
**C40**	0.33	154	341	47	80	712	1068	1.2
**C50**	0.29	152	398	42	84	639	1135	1.2
**C60**	0.25	148	432	59	101	650	1060	1.2

**Table 4:** Design scheme of four factors and three levels.

NO.	Level Combination	Experimental Condition			
		Cement	Water Reducing Agent	Sand	Stone
1	A_1_B_1_C_1_D_1_	A_1_	B_1_	C_1_	D_1_
2	A_1_B_2_C_2_D_2_	A_1_	B_2_	C_2_	D_2_
3	A_1_B_3_C_3_D_3_	A_1_	B_3_	C_3_	D_3_
4	A_2_B_1_C_2_D_3_	A_2_	B_1_	C_2_	D_3_
5	A_2_B_2_C_3_D_1_	A_2_	B_2_	C_3_	D_1_
6	A_2_B_3_C_1_D_2_	A_2_	B_3_	C_1_	D_2_
7	A_3_B_1_C_3_D_2_	A_3_	B_1_	C_3_	D_2_
8	A_3_B_2_C_1_D_3_	A_3_	B_2_	C_1_	D_3_
9	A_3_B_3_C_2_D_1_	A_3_	B_3_	C_2_	D_1_

**Table 5:** Evaluation criteria for surface voids of fair-faced concrete.

Rating	Excellent	Good	Unqualified
Evaluation	Maximum diameter is not greater than 4 mm, and the surface porosity area ratio is not more than 0.2%.	Maximum diameter is greater than 4 mm but less than or equal to 8 mm, and the surface porosity area ratio is not more than 0.2%	The surface voids area ratio exceeds 0.2%.

**Table 6:** The GPC results of P(MAH-MPEG_n_MA)_m_ superplasticizers.

Entry	Feed Ratio (MAH:MPEG_n_MA)	Superplasticizer P(MAH-MPEG_n_MA)_m_	GPC Result		
			Mn/g·mol^−1^	Mw/g·mol^−1^	PDI
A	3.5:1	P(MAH_3.5_-MPEG_16_MA)_5_	5500	6800	1.25
B	4:1	P(MAH_4_-MPEG_16_MA)_9_	10,600	15,200	1.42
C	4.5:1	P(MAH_4.5_-MPEG_16_MA)_12_	15,200	21,100	1.38
D	4:1	P(MAH_4_-MPEG_45_MA)_9_	22,000	31,200	1.41
E	4:1	P(MAH_4_-MPEG_67_MA)_9_	30,100	41,800	1.39

**Table 7:** Effect of P(MAH-MPEG_n_MA)_m_ on cement paste fluidity.

Entry	Superplasticizer P(MAA-MPEG_n_MA)_m_	Dosage/%	Fluidity/mm		
			Initial	0.5 h	1 h
A	P(MAA_3.5_-MPEG_16_MA)_5_	0.18	203	185	163
B	P(MAA_4_-MPEG_16_MA)_9_		230	205	198
C	P(MAA_4.5_-MPEG_16_MA)_12_		222	171	148
D	P(MAA_4_-MPEG_45_MA)_9_		220	190	178
E	P(MAA_4_-MPEG_67_MA)_9_		205	170	139

**Table 8:** Mix proportions of SCC for determining the dosage of P(MAH-MPEG_n_MA)_m_ (kg·m^−3^).

Entry	Cement	Sand	Coarse Aggregate	P(MAH- MPEG_n_MA)_m_	VEA	Air-Entraining Agent	Water
1	520	826	894	3.12	1.04	0.052	165
2	520	826	894	4.16	1.04	0.052	165
3	520	826	894	5.20	1.04	0.052	165
4	520	826	894	6.24	1.04	0.052	165

**Table 9:** Influence of the dosage of P(MAA-MPEG_n_MA)_m_ on the flow ability of SCC.

Entry	P(MAH-MPEG_n_MA)_m_	W/%	V Funnel/s	T_50_/s	Slump Flow/mm	Segregation
1	P(MAH_3.5_-MPEG_16_MA)_5_	0.6	17	-	360	No
2	P(MAH_3.5_-MPEG_16_MA)_5_	0.8	13	12	540	No
3	P(MAH_3.5_-MPEG_16_MA)_5_	1.0	11	11	580	No
4	P(MAH_3.5_-MPEG_16_MA)_5_	1.2	10	9	600	No
5	P(MAH_4_-MPEG_16_MA)_9_	0.6	9	10	610	No
6	P(MAH_4_-MPEG_16_MA)_9_	0.8	8.2	8	650	No
7	P(MAH_4_-MPEG_16_MA)_9_	1.0	6.5	3.8	700	No
8	P(MAH_4_-MPEG_16_MA)_9_	1.2	7.6	3.5	720	Yes
9	P(MAH_4.5_-MPEG_16_MA)_12_	0.6	10	8.6	610	No
10	P(MAH_4.5_-MPEG_16_MA)_12_	0.8	8.5	7.7	630	No
11	P(MAH_4.5_-MPEG_16_MA)_12_	1.0	7	6.9	650	No
12	P(MAH_4.5_-MPEG_16_MA)_12_	1.2	7.8	5.4	670	No
13	P(MAH_4_-MPEG_45_MA)_9_	0.6	10	8	600	No
14	P(MAH_4_-MPEG_45_MA)_9_	0.8	9	6.5	640	No
15	P(MAH_4_-MPEG_45_MA)_9_	1.0	8.9	5.2	660	No
16	P(MAH_4_-MPEG_45_MA)_9_	1.2	7.6	47	680	Some mortar separated out
17	P(MAH_4_-MPEG_67_MA)_9_	0.6	16	13	540	No
18	P(MAH_4_-MPEG_67_MA)_9_	0.8	13	10	570	No
19	P(MAH_4_-MPEG_67_MA)_9_	1.0	10	8.6	610	No
20	P(MAH_4_-MPEG_67_MA)_9_	12	8.6	7.5	630	No

## Data Availability

Data are contained within the article.
